# Protective effect of Suxiao jiuxin pill, a traditional Chinese medicine, against acute myocardial ischemia in dogs

**DOI:** 10.1186/s12906-015-0908-9

**Published:** 2015-10-19

**Authors:** Zhiqiang Lu, Yanjun Zhang, Pengwei Zhuang, Jinbao Zhang, Huifang Zhou, Mixia Zhang, Xinpeng Yang, Jinlei Wang, Dan Liu, Yongling Tong

**Affiliations:** Chinese Materia Medica College, Tianjin University of Traditional Chinese Medicine, Tianjin, China; Tianjin State Key Laboratory of Modern Chinese Medicine, Tianjin University of Traditional Chinese Medicine, Tianjin, China; Department of Experimental Teaching, Tianjin University of Traditional Chinese Medicine, Tianjin, China; The Sixth Chinese Drugs Factory of Tianjin Zhongxin Pharmaceutical Co., Ltd., Tianjin, China

## Abstract

**Background:**

The purpose of this study was to investigate the effect of Suxiao Jiuxin Pill (SX), a traditional Chinese medicine, on acute myocardial ischemia induced by coronary occlusion in anesthetized dogs.

**Methods:**

Acute myocardial ischemia model was established by ligating the left anterior descending artery to reduce flow by 90 %. Adult mongrel dogs were randomly divided into six groups: model, SX high dose, SX middle dose, SX low dose, Isosorbide dinitrate (ISD) and Sham groups. Adult mongrel dogs were anesthetized and instrumented for measurements of heart rate (HR), mean arterial pressure (MAP), left ventricular systolic pressure (LVSP), left ventricular dP/dt, coronary blood flow (CBF), myocardial blood flow (MBF), coronary vascular resistance (CVR), and epicardial electrocardiogram (EECG). After administration with SX, changes in hemodynamics were recorded. Serum enzymes and blood gas analysis were also detected.

**Results:**

SX has a dose-dependent effect on the reduction of infarct size. Besides, SX exerted a notable inhibition on the elevation of serum creatine kinase MB (CK-MB), lactate dehydrogenase (LDH), malondialdehyde (MDA), and elevation in the superoxide dismutase (SOD) activity. SX also showed a capacity to recover myocardial function by significantly reducing MAP, CVR, LVSP, left ventricular systolic pressure (LVEDP), systolic blood pressure (SBP), diastolic blood pressure (SDP), and increasing CBF and myocardial blood flow (MBF). In addition, SX high dose group markedly reduced total mV of ST segment elevation (Σ-ST), total number of sites with this degree of ST segment elevation (N-ST) and oxygen extraction ratio (O_2_ Extr).

**Conclusion:**

SX can improve hemodynamic and myocardial oxygen metabolism, reduce the degree and scope of myocardial ischemia, and hence exert notable anti-anginal ischaemic effect.

## Background

Cardiovascular disease is the leading cause of death worldwide, affecting not only high-income but also low- and middle-income countries. Coronary heart disease (CHD) and ischemic cardiomyopathy are the most important types of cardiovascular disease [1, 2]. Modern medicine has improved dramatically as the treatment of cardiovascular disease. Primary therapies such as antianginal drugs and anticonvulsive drug are the first-line treatments for cardiovascular disease [3]. At the same time, some of the traditional Chinese medicine (TCM) gradually entered into public view because of its remarkable clinical effect. Traditional Chinese medicine has about 2000 to 3000 years history with unique theories for concepts of etiology and systems of diagnose and cure illness. Chinese medicines have many clinical applications in treatment of cardiovascular disease, such as Suxiao jiuxin pill (SX) for Angina Pectoris [4–6].

SX is one of the most efficacious Chinese patent medicines for the treatment of cardiocerebral vascular diseases. It was developed on years of clinical experience by old Chinese medicine experts – Chenggui Zhang in the 1980s, and manufactured by the Sixth Chinese Drugs Factory of Tianjin Zhongxin Pharmaceutical Co., Ltd., Tianjin, China [7]. It has been used for the treatment of coronary heart disease, angina pectoris, myocardial infarction, and hence added on the national essential drug list of China with 30 years of clinical application [8]. SX has been shown to rapid remission of angina pectoris and improve its symptoms with small doses in addition there is no obvious discomfort, side effects and drug resistance [9]. In China, many people who suffer from angina pectoris take SX to prevent and treat angina pectoris. Recent studies suggest that SX was shown to significantly improve myocardial ischaemia and reduce the incidence of myocardial infarction by improving microcirculation, reducing the blood viscosity, increasing coronary arterial blood flow, dilating coronary vessels and improving myocardial blood supply [10–13]. However, despite many years of clinical experience in the use of anti-angina, the corresponding basic research was relatively scarce.

Ligusticum Chuanxiong (*Rhizoma Chuanxiong*) and Borneol (*Borneolum Syntheticum*) are the two main components of SX [9]. *Ligusticum Chuanxiong* is a popular Chinese medicinal plant with the effect of blood and Qi circulation, chills and pain relief, and clear gloomy dampness in traditional Chinese medicine for thousands of years [14]. Tetramethylpyrazine, a bioactive ingredient contained in Ligusticum Chuanxiong, is broadly applied in the treatment of vascular diseases in China, e. g. myocardial and cerebral infarction [15]. The average content of tetramethylpyrazine is 0.04084 mg/pill in SX [16]. Borneol is another major component of SX and essential oil in many other medicinal plants, as well as a popular traditional Chinese medicine [17]. Report showed that borneol shares many bioactivities such as anti-inflammatory, antithrombotic, and protection against cerebral ischemic damage [18].

In this study, we utilize the myocardial ischemia model, ligating left anterior descending (LAD), to investigate the anti-ischemic effect of SX on hearts. Myocardial infarct size, blood gas variables, hemodynamic and epicardial electrocardiogram, and serum levels of creatine kinase MB (CK-MB), lactate dehydrogenase (LDH), malondialdehyde (MDA), and superoxide dismutase (SOD), parameters were measured during the experiment.

## Methods

### Ethics statement

This study was carried out in strict accordance with the recommendations in the Regulations on the management of laboratory animal [19]. All animal procedures were performed in accordance with the Animal Ethics Committee of Tianjin University of Traditional Chinese Medicine (TCM-2012-011-E05).

### Animal

Healthy adult male mongrel dogs (12–18 kg) were used in this study provided by Beijing Keyu animal breeding center, and purchased in Tianjin University of Traditional Chinese Medicine Experimental Animal Center after ethical and scientific approval. All dogs were kept and maintained under laboratory conditions of temperature, humidity, and were allowed free access to food and water. After one week, 44 healthy dogs were selected by weight and ECG (the dogs with unqualified weight and ECG were eliminated), and randomly divided into six groups based on weight: model (saline, 1 ml/kg), SX high dose (SX, 25.60 mg/ml/kg), SX middle dose (SX, 12.80 mg/ml/kg), SX low does (SX, 6.40 mg/ml/kg,) and Isosorbide dinitrate (ISD, 0.80 mg/ml/kg), and Sham (saline, 1 ml/kg) with 7, 7, 9, 7, 6, and 8 animals in each groups respectively. Animals that died during the experiment were not included. SX (40 mg/tablet) were kindly supplied by the Sixth Chinese Drugs Factory of Tianjin Zhongxin Pharmaceutical Co., Ltd.

### Surgical preparation

This study was performed using the experimental model of acute myocardial ischemia (AMI). Dogs were fasted overnight prior to the surgery, but allowed free access to water. A previously described model of open chest anesthetized dogs was used [20, 21]. Dogs were anesthetized with 30 mg/kg of i.v. sodium pentobarbital (St. Louis, MO, USA), and additional doses were given as needed throughout the experiment. Aseptic surgical technique was performed, and the dog’s body kept at a constant temperature by thermostatic operating table. The dogs were intubated and ventilated by a respirator (DHX-500, Chengdu of China) with room air (tidal volume, 200 ml/kg per min; respiratory rate, 18 cycles/min). A polyethylene catheter was inserted into the venous sinus through the right external jugular vein for venous blood gas analysis and blood samples. The left femoral artery was connected with a polyethylene catheter for arterial blood gas analysis and blood pressure measurement. An 8 to 10 cm thoracotomy was performed in the left fifth intercostal space and the heart was suspended in a pericardial cradle. The left circumflex coronary artery (LCX) was isolated 3–5 mm from the origin and an ultrasonic transit-time perivascular flow probe (2.5 or 3.0 mm, Transonic Systems Inc., Ithaca, NY, USA) was placed around LCX to measure coronary blood flow (CBF), as described recently [22]. A segment of left anterior descending coronary artery (LAD) was isolated between the first and second diagonal branch and the blood flow were mesured by the same method as above. A double 7.0 silk ligature was passed through the LAD for latter two-stage occlusion. The double silk ligature was cut, thus becoming two ligatures. The first ligature was tightened to reduce flow of LAD by 50 % for 10 min, and then the second ligature was tightened with needle to decreases the flow by 90 % [23]. Sham-operated dogs were subjected to the same procedures without LAD ligation. An infusion tube was inserted into duodenum for drug administration after occlusion. Thirty minutes after LAD ligation, all dogs were injected through the infusion tube with drugs or saline.

### Experimental procedure

#### Hemodynamic measurements

Coronary angiography catheter (7F) was inserted via the right femoral artery into the left ventricle by the Seldinger technique and then connected to the MP150 analogue-to-digital converter (Biopac Systems, Inc., Goleta, CA). The heart rate (HR), mean arterial pressure (MAP), left ventricular systolic pressure (LVSP), left ventricular end-diastolic pressure (LVEDP), the maximum and minimum first derivative of LVSP (+dP/dtmax and -dP/dtmax) were measured. Moreover a polyethylene catheter was inserted to left femoral artery for the measurement of the systolic blood pressure (SBP), diastolic blood pressure (DBP), and mean arterial pressure (MAP). The CBF were measured by an ultrasonic transit-time perivascular flow probe and recorded by MP150. Myocardial blood flow (MBF, in milliliters per minute per 100 g) was approximately calculated from the equation: MBF = CBF × 300/Cardiac Weight [24]. Coronary vascular resistance (CVR) was calculated by dividing mean arterial pressure (MAP) with the respective MBF [25]. Hemodynamic data were collected at pre-occlusion, pre-administration, and at all time in 180 min after administration.

#### Determination of epicardial ECG

Epicardial electrocardiogram (EECG) with 30 unipolar silver wire electrodes, connected to MP150 were placed on the anterior surface of the left ventricle. Characteristic electrical parameters such as ECG ST elevations were recorded from points of a 5 × 6 matrix on the anterior epicardial surface [26]. All EECG data were analyzed using the PowerLab software system (Chart version 7.3.7, AD Instruments). The determination of epicardial ECG was measured at pre-occlusion, pre-administration, and 30, 60, 90, 120, 180 min after administration. A ST segment elevation of more than 2 mV was regarded as the ischemic criteria to calculate the degree of myocardial ischemia (total mV of ST segment elevation, Σ-ST) and the scope of myocardial ischemia (total number of sites with this degree of ST segment elevation, N-ST) [27]. All hemodynamic and cardiodynamic data were sampled by a laboratory computer at 1000 Hz, and mean values for 10 cardiac cycles were saved on the laboratory computer for future analysis.

#### Serum enzymes activities and blood gas analysis

The blood samples were collected from the venous sinus and test for CK-MB, LDH, MDA and SOD levels by the diagnostic kits (Nanjing Jiancheng Bioengineering Institute, China) with recommended protocol. The venous sinus blood and arterial blood were sampled anaerobically in heparinized syringes and immediately analyzed for analysis of oxygen tensions (PO_2_) and oxygen saturation (SO_2_) by a blood gas analyzing machine (Radiometer ABL5, Denmark). Oxygen content of arterial (CaO_2_) and venous (CvO_2_) blood in ml/dl was calculated as follows [28]: CaO_2_ = (SaO_2_ × Hgb × 1.34) + (0.003 × PaO_2_); CvO_2_ = (SvO_2_ × Hgb × 1.34) + (0.003 × PvO_2_); oxygen extraction ratio (O_2_ Extr) = (CaO_2_ − CvO_2_)/CaO_2_; myocardial oxygen consumption (MVO_2_) = CBF×(CaO_2_ − CvO_2_), where PaO_2_ is arterial carbon dioxide tension, PvO_2_ is venous sinus oxygen tension, SaO_2_ is arterial oxygen saturation, Hgb is hemoglobin, SvO_2_ is venous sinus oxygen saturation. All blood samples were measured at pre-occlusion, pre-administration, and 30, 60, 90, 120, 180 min after administration.

#### Determination of infarct size

After 180 min of occlusion, the heart was removed and the atrium was discarded. The ventricle was quickly washed with normal saline, placed at −20 °C for 30 min, cut into 5 equal portions, and then placed in 1 % triphenyl tetrazolium chloride (TTC) solution in phosphate buffer for 10 min. After TTC staining, the viable myocardium was stained red and necrotic myocardium remained pale. Ischemic zone and non-ischemic zone were weighed. The weights of each ischemic area were calculated using weight of the ischemic zone/total left ventricular weight × 100 % as reported previously [29].

### Statistical analysis

The data were expressed as mean ± S.D., a two-factor repeated-measures analysis of variance (ANOVA) and paired-test were performed using SPSS 16.0 statistical software. The *P*-values <0.05 were considered to be statistically significant.

## Results

### Effect of SX on infarct size

**Fig. 1:**
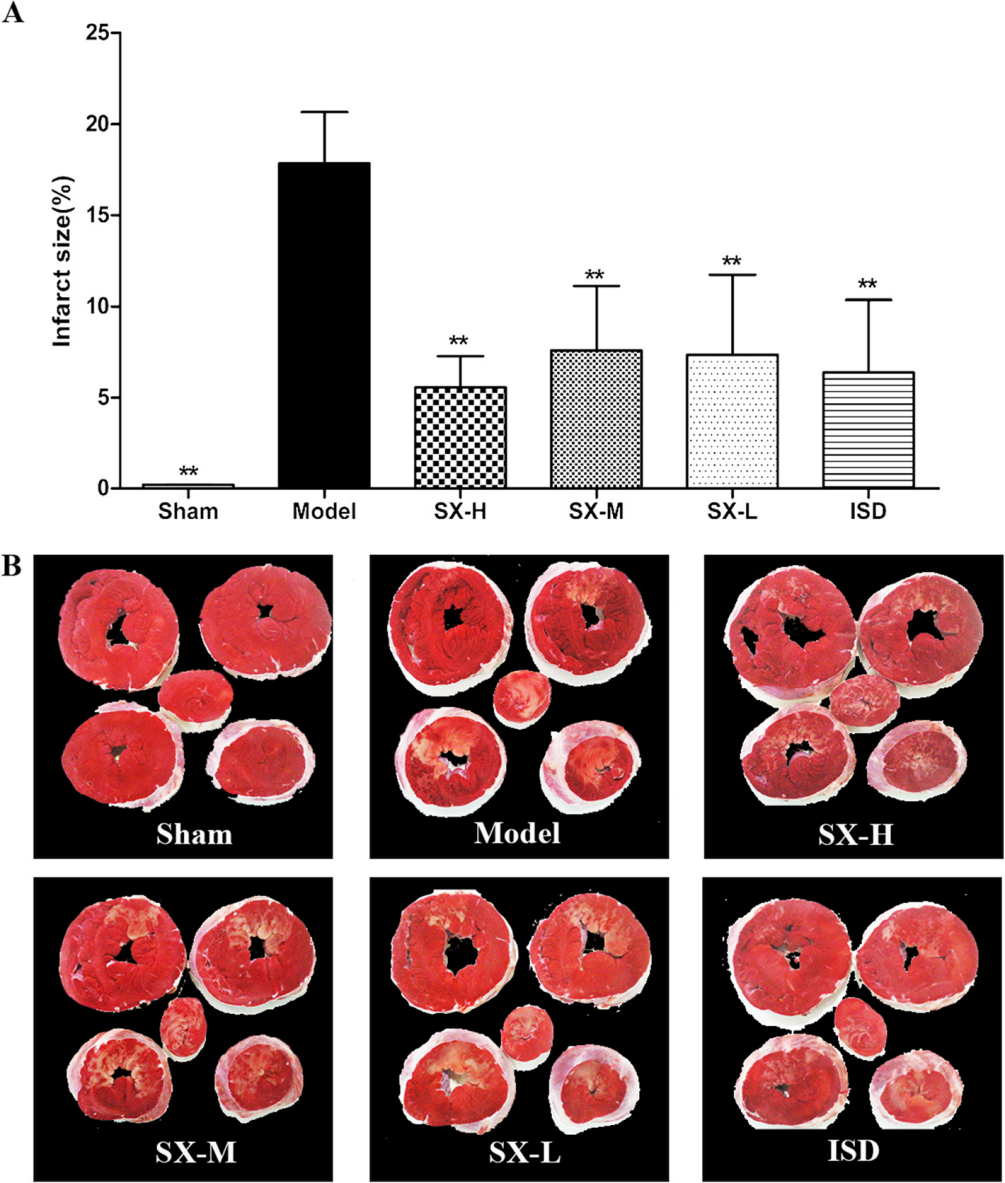
Effects of SX on infarct size (*upper panel*, **a**) in dog hearts subjected to 180 min of ischemia. Each *bar* shows mean ± S.D. **P < 0.01 vs. model group. Representative heart morphological photographs of infarct zone are shown on the *lower panel* (**b**). *White* areas indicate infarcted tissues stained by TTC

To compare the visually observed effect of SX on anti-ischemic, the infarct size of the ischemic hearts were analysed. Figure 1 shows the percentage of infarct size of hearts subjected to 180 min of ischemia. Post-ischemia myocardium was associated with a 17.84 ± 2.83 % infarct size which was consistently reduced by increasing concentrations of SX to 7.33 ± 4.39 % (*P* < 0.01, 6.40 mg/kg SX), 6.03 ± 3.13 % (P <0.01, 12.80 mg/kg SX), and 5.55 ± 1.72 % (*P* <0.01, 25.60 mg/kg SX), in low, middle, and high dose group respectively (Fig. 1). The same effect was found in the ISD group which show no significant difference with SX (25.60 mg/kg) group. The lower panel in Fig. 1b shown the infarcted (white) and viable (red) tissue, and the infract areas treated with SX show unequivocal signs of improvement changed from whole white to red alternating with white. There were significant differences between the model group and SX groups.

### Effect of SX on the degree and scope of myocardial ischemia

**Fig. 2:**
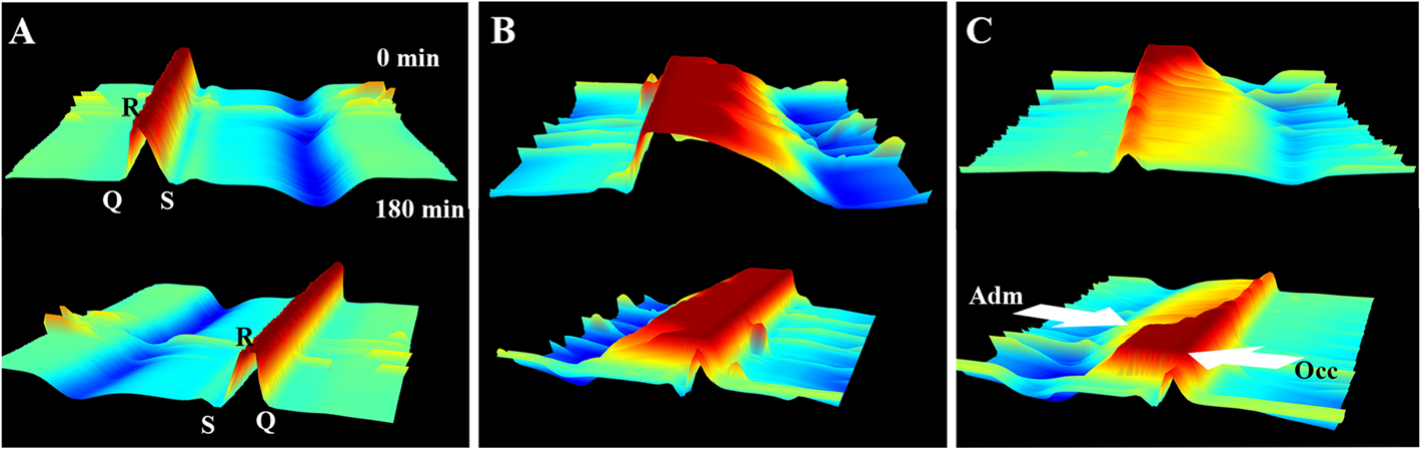
Changes of epicardial ECG waveform after administration of SX in dogs. **a**, **b** and **c** represented epicardial ECG waterfall plot of sham, model and SX high dose (25.6 mg/kg) group. Continuous epicardial ECG monitoring revealed that ST segment elevated after occlusion and depressed after treatment SX

Epicardial electrocardiogram was checked to compare the degree and scope of myocardial ischemia during the experiment. In the model group, the Σ-ST and N-ST were significantly increased from −18.85 ± 11.09 mV to 228.78 ± 122.39 mV, and 0.33 ± 0.52 points to 24.67 ± 7.47 points respectively (Tables 1 and 2). Following treatment with SX and ISD, those indexes were significantly decreased at 180 min compared with the model group (*P* < 0.05). Additionally the Σ-ST and N-ST of dogs treated with high dose SX was significantly reduced by 69.18 % (from 239.47 ± 111.84 mV to 73.81 ± 69.97 mV, *P* < 0.05), and 26.75 % (from 27.33 ± 2.25 points to 15.67 ± 6.56 points, *P* < 0.01), respectively (Tables 1 and 2). After occlusion, epicardial ECG changed dramatically and ST segment elevated significantly (Fig. 2b). However, the higher dose (25.6 mg/kg) of SX caused significant depression of ST segment on the epicardial ECG (Fig. 2c).

**Table 1:** The degree of myocardial ischemia (Σ-ST)

Group	Dose mg/kg	Pre-Occ (mV)	Pre-Adm (mV)	Post-Adm (mV)				
				30	60	90	120	180
Sham		−35.47 ± 7.80	−31.40 ± 12.07	−28.14 ± 22.35	−34.21 ± 18.71	−31.54 ± 23.80	−32.10 ± 15.66	−25.93 ± 13.07
Model		−18.85 ± 11.09	228.78 ± 122.39	209.86 ± 108.56	187.76 ± 74.35	182.10 ± 68.97	184.33 ± 71.08	189.54 ± 84.49
SX	25.60	−30.30 ± 36.01	239.47 ± 111.84	156.48 ± 127.59&	121.72 ± 93.09&	116.66 ± 76.64	94.62 ± 54.57*&	73.81 ± 69.97*&
SX	12.80	−39.02 ± 32.56	242.85 ± 70.06	155.22 ± 140.79&	144.12 ± 114.97&	147.20 ± 101.80&	135.71 ± 103.29&	98.11 ± 95.72*&&
SX	6.40	−43.29 ± 49.84	212.47 ± 72.40	127.46 ± 58.49&	130.03 ± 73.24&	146.50 ± 85.40&	132.43 ± 103.90&	140.03 ± 127.08&&
ISD	0.8	−20.17 ± 14.69	199.2 ± 92.22	122.5 ± 65.63	119.43 ± 64.96	107.89 ± 81.53	96.51 ± 85.91&	83.28 ± 79.61*&

All data were expressed as mean ± S.D. *P < 0.05, **P < 0.01 vs. model group; & *P* < 0.05, && *P* < 0.01 vs. Pre-Adm

**Table 2:** The degree of myocardial ischemia (N-ST)

Group	Dose mg/kg	Pre-Occ (mV)	Pre-Adm (mV)	Post-Adm (mV)				
				30	60	90	120	180
Sham		0.83 ± 1.60	0.17 ± 0.41	0.17 ± 0.41	0.33 ± 0.52	0.17 ± 0.41	0.50 ± 1.22	0.00 ± 0.00
Model		0.33 ± 0.52	24.67 ± 7.47	25.83 ± 5.78	26.33 ± 5.24	26.67 ± 5.13	26.00 ± 5.51	26.67 ± 3.56
SX	25.60	1.33 ± 1.86	27.33 ± 2.25	21.33 ± 4.32&	17.33 ± 8.55&	17.33 ± 7.99*&	16.17 ± 7.47*&&	15.67 ± 6.56**&&
SX	12.80	0.86 ± 0.90	25.86 ± 3.08	16.57 ± 7.74&&	16.71 ± 6.99*&&	18.57 ± 4.96*&	18.71 ± 5.94*&	16.43 ± 6.68**&&
SX	6.40	1.83 ± 3.13	25.83 ± 2.32	19.67 ± 4.68&	19.17 ± 6.46&	19.83 ± 5.81&	18.83 ± 6.15&	18.67 ± 6.47*&
ISD	0.8	1.67 ± 1.97	25.33 ± 5.72	21.17 ± 6.82	21 ± 6.75	19 ± 9.19	15.83 ± 9.95&	16.33 ± 9.05*&

All data were expressed as mean ± S.D. *P < 0.05, **P < 0.01 vs. model group; & *P* < 0.05, && *P* < 0.01 vs. Pre-Adm

### Effect of SX on hemodynamic

Hemodynamic monitoring is one of the most important methods to measure and interpret the performance of the cardiovascular system. So the effects of SX on hemodynamic were checked in the present study. And the result shown no significant differences between all the six groups at pre-occlusion (*P* > 0.05) (Table 3). Moreover no significant differences were observed after treatment with regard to HR and ± dP/dt (*P* > 0.05). Compared with model group, administration with SX high dose and ISD caused a significant decrease in MAP (by 15.05 and 13.92 %, respectively), and in LVEDP (by 31.40 and 28.30 %) (*P* < 0.05). The LVSP was significantly decreased in high, middle, low SX, and ISD group at 180 min post-administration (*P* < 0.01). In the model group, CBF, MBF decreased, and CVR were increased significantly (*P* < 0.05), however, the trend were significantly reversed in the dogs treated with SX and ISD showed in Table 3 (*P* < 0.05).

**Table 3:** Hemodynamic parameters at pre-occlusion, pre-administration and post-administration (180 min)

Time	Parameters								
	HR	MAP	LVSP	LVEDP	+dP/dtmax	-dP/dtmix	CBF	MBF	CVR
	bpm	mmHg	mmHg	mmHg	mmHg/s	mmHg/s	ml/min	ml/min.100 g	mmHg.min/mL
Pre-Occ									
Sham	175.35 ± 31.3	119.18 ± 14.43	147.27 ± 23.14	4.89 ± 3.36	3050.63 ± 542.34	−2610.66 ± 670.12	45.19 ± 2.41	161.31 ± 32.34	0.74 ± 0.09
Model	170.03 ± 35.3	123.88 ± 14.1	149.15 ± 13.74	3.45 ± 8.2	2850.71 ± 600.93	−2587.09 ± 477.81	47.86 ± 8.87	182.79 ± 60.94	0.79 ± 0.23
SX-H	167.88 ± 23.46	106.99 ± 21.01	135.26 ± 26.29	4.6 ± 4.2	2987.07 ± 825.57	−2329.83 ± 731.73	45.89 ± 7.32	163.02 ± 32.79	0.78 ± 0.21
SX-M	171.5 ± 25.06	106.92 ± 15.12	139.34 ± 7.64	3.68 ± 3.33	2764.46 ± 355	−2426.34 ± 459.77	44.05 ± 6.15	172.57 ± 43.87	0.76 ± 0.24
SX-L	167.4 ± 24.96	113.47 ± 16.49	136.85 ± 17.57	5.1 ± 2.21	2785.09 ± 635.7	−2533.04 ± 765.36	46.07 ± 13.52	145.87 ± 50.5	0.82 ± 0.25
ISD	171.16 ± 18.55	118.1 ± 20.7	146.88 ± 24.73	4.94 ± 1.9	2797.02 ± 481.73	−2328 ± 542.84	46.84 ± 2.81	185.2 ± 50.47	0.72 ± 0.18
Pre-Adm									
Sham	175.97 ± 27.68	121.45 ± 16.64	149.37 ± 25.47	5.15 ± 0.53	3222.04 ± 595.88	−2720.74 ± 912.21	49.28 ± 7.95	171.94 ± 12.13	0.71 ± 0.09
Model	163.34 ± 33.96	104.26 ± 1.6	134.8 ± 10.66	7.69 ± 3.1	2445.67 ± 343.97	−2416.3 ± 310.43	44.64 ± 10.78	179.61 ± 55.94	0.74 ± 0.19
SX-H	160.02 ± 23.66	97.23 ± 18.85	131.07 ± 22.75	7.74 ± 3.25	2234.54 ± 642.44	−2110.49 ± 636.37	40.79 ± 8.1	149.81 ± 28.52	0.67 ± 0.19
SX-M	172.79 ± 26.4	102.51 ± 19.92	133.58 ± 9.97	6.81 ± 2.39	2549.35 ± 364.23	−2373.87 ± 334.64	44.68 ± 10.48	174.87 ± 58.35	0.65 ± 0.22
SX-L	165.27 ± 23.43	103.91 ± 15.53	125.56 ± 14.96	8.1 ± 3.1	2437.5 ± 482.9	−2308.27 ± 542.33	44.02 ± 12.37	137.31 ± 34.7	0.75 ± 0.18
ISD	165.43 ± 20.48	101.5 ± 23.81	112.53 ± 11.88	8.91 ± 1.35	2235.42 ± 509.53	−2037.27 ± 511.09	46.67 ± 10.35	175.73 ± 77.42	0.70 ± 0.16
Post-Adm									
Sham	176.98 ± 33.58	111.3 ± 18.19	133.38 ± 18.78	4.7 ± 2.6	2830.94 ± 814	−2595.17 ± 718.67	50.37 ± 10.36	174.92 ± 18.42	0.68 ± 0.1
Model	168.9 ± 38.59	95.3 ± 12.26	132.34 ± 9.95	8.94 ± 1.87	1873.17 ± 532.67	−2120.28 ± 544.01	33.15 ± 9.75&	126.63 ± 27.83&	0.82 ± 0.19&
SX-H	184.27 ± 37.12	80.96 ± 5.29*	96.57 ± 12.05**&&	5.82 ± 2.65*&	1835.97 ± 141.59	−1862.88 ± 265.58	58.86 ± 13.3**&	219 ± 64.55*&&	0.36 ± 0.09**&&
SX-M	186.38 ± 36.32	90.9 ± 16.36	97.23 ± 22.2**&&	5.5 ± 3.05*	1946.72 ± 380.59	−1939.73 ± 720.86	49.66 ± 8.29*	205.25 ± 74.85*&&	0.48 ± 0.21*&
SX-L	174.25 ± 20.49	96.5 ± 17.19	109.33 ± 14.06**	6.09 ± 3.2*	2183.55 ± 423.96	−2081.71 ± 441.56	52.13 ± 13.41*	180.23 ± 53.75*&	0.55 ± 0.31&
ISD	167.28 ± 22.23	82.03 ± 2.51*	113.51 ± 6.79**	6.41 ± 3.19*&	2008.15 ± 175.04	−2024.36 ± 265.35	61 ± 13.55**&	222.55 ± 93.78*&&	0.4 ± 0.15**&&

All data were expressed as mean ± S.D. *HR* heart rate, *MAP* mean arterial pressure, *LVSP* left ventricular systolic pressure, *LVEDP* left ventricular end-diastolic pressure, +*dP*/*dtmax* maximal positive left ventricular dP/dt, -*dP*/*dtmix* minimal positive left ventricular dP/dt, *CBF* coronary blood flow, *MBF* myocardial blood flow, *CVR* coronary vascular resistance. *P < 0.05, **P < 0.01 vs. model group; & *P* < 0.05, && *P* < 0.01 vs. Pre-Adm

### Effect of SX on the serum CK-MB, LDH, MDA and SOD activity

**Fig. 3:**
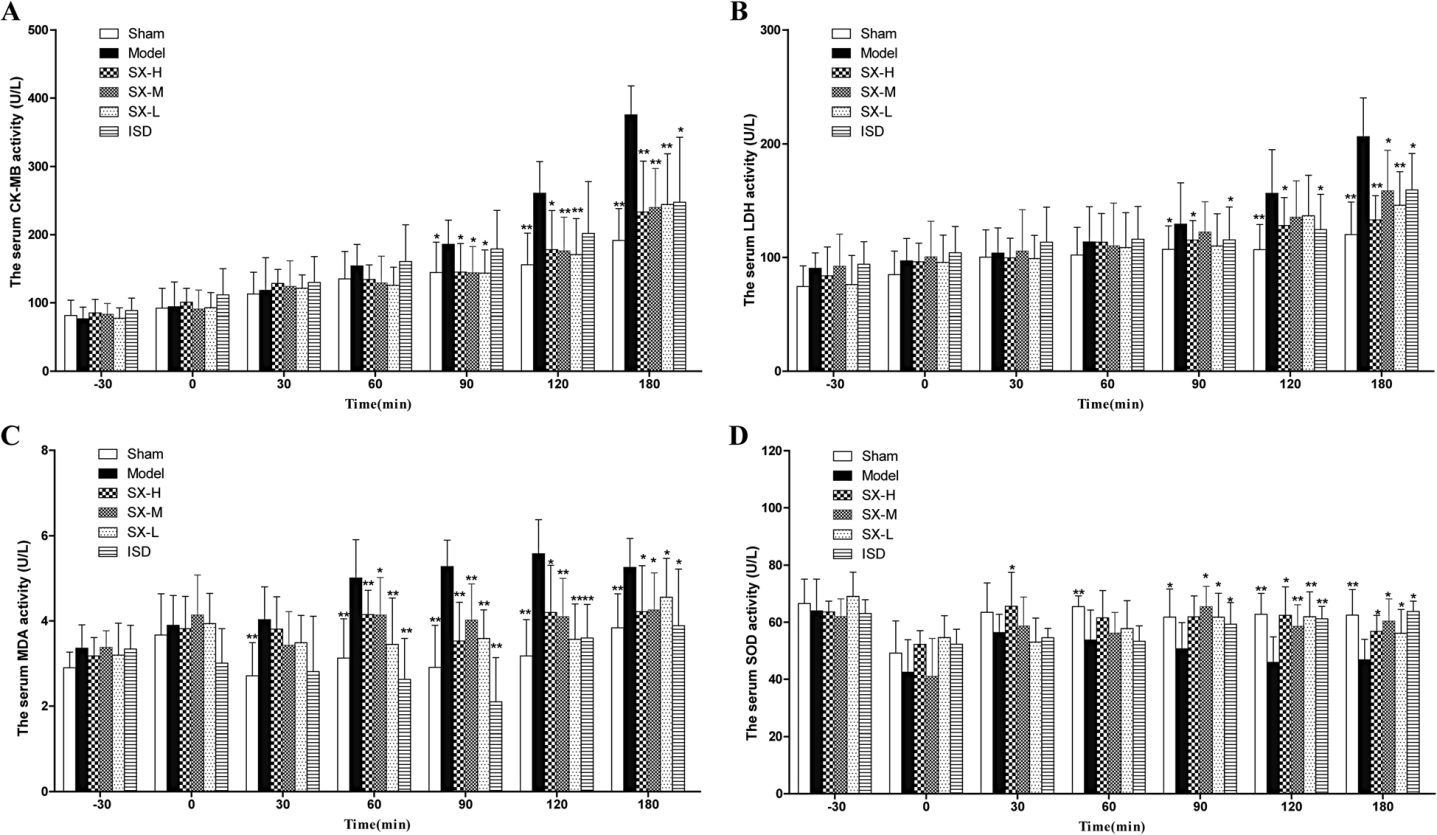
Effects of SX on the serum CK-MB, LDH, MDA and SOD activity corresponding to (**a**, **b**, **c** and **d**). Serum enzyme indicators were detected at −30 min (baseline), at 0 min (pre-administration), and at 30, 60, 90, 120, 180 min in post-administration. *Adm* Administration, *Occ* Occlusion. Each *bar* shows mean ± S.D. *P < 0.05, **P < 0.01 vs. model group

The changes of the levels of serum cardiac enzymes were confirmed during the acute myocardial ischemia. In the present study, the effects of SX on CK-MB, LDH, MDA, and SOD were checked (Fig. 3). Treatment with all the three SX doses and ISD significantly reduced serum CK-MB, LDH, MDA, and increased SOD levels in 90–180 min of post-occlusion compared with the model group (*P* < 0.05, Fig. 3a, b, c, d). These results suggested that SX could reduce lipid peroxidation and increase superoxide dismutase resulting in the protection against the myocardial ischemic damage.

### Effect of SX on blood gas measurements

The blood gas analysis could reflect the oxygen metabolism and oxygen consumption during acute myocardial ischemia. Blood gas data of the six groups were shown in Table 4. Compare with model group, a slight increase in CaO_2_ was observed in groups treated with SX and ISD, but it was not statistically significant (*P* > 0.05). Besides, there were no differences in CvO_2_ in all groups. After occlusion, the O_2_ extraction was increase gradually in all group, the increase trend in SX (25.60 mg/kg) and ISD group was significantly lower than model group (*P* < 0.05), and the same trend were observed in MVO_2_, but this change was not statistically significant compared with model group.

**Table 4:** Blood gas analysis parameters at pre-occlusion, pre-administration and post-administration (180 min)

	Group	Pre-Occ	Pre-Adm	Post-Adm (min)				
				30	60	90	120	180
CvO_2_ (vol %)	Sham	11.01 ± 4.84	11.3 ± 4.26	10.93 ± 4.44	8.73 ± 4.41	8.67 ± 3.81	8.05 ± 3.53	7.88 ± 3.27
	Model	11.38 ± 2.8	10.36 ± 4.1	9.24 ± 4.19	7.99 ± 3.64	6.57 ± 3.3	6.57 ± 3.35	5.43 ± 3.01
	SX-H	12.06 ± 3.2	11.32 ± 4.36	10.47 ± 2.44	9.64 ± 1.95	8.38 ± 1.68	8.12 ± 2.29	7.38 ± 2.55
	SX-M	12.67 ± 3.63	12.01 ± 3.8	10.76 ± 4.14	9.99 ± 4.27	8.94 ± 4.19	8.94 ± 4.66	8.35 ± 5.17
	SX-L	12.01 ± 2.82	9.53 ± 3.37	8.5 ± 3	8.81 ± 4.2	8.64 ± 3.31	7.47 ± 2.27	5.96 ± 2.73
	ISD	11.94 ± 1.21	11.44 ± 1.34	11.51 ± 2.16	10.31 ± 1.77	9.95 ± 2.65	9.78 ± 3.03	8.52 ± 3.42
CaO_2_ (vol %)	Sham	19 ± 0.68	19.21 ± 0.41	18.99 ± 0.45	18.81 ± 0.47	18.82 ± 0.41	18.68 ± 0.64	18.74 ± 0.73
	Model	18.78 ± 1.2	18.32 ± 2.32	17.83 ± 1.02	17.4 ± 1.8	17.34 ± 2.04	17.14 ± 2.19	16.86 ± 2.07
	SX-H	19 ± 0.38	18.64 ± 0.53	18.46 ± 0.69	18.31 ± 0.78	18.2 ± 0.85	18.2 ± 0.58	17.81 ± 1.17
	SX-M	18.82 ± 0.43	18.54 ± 0.78	18.42 ± 0.88	18.22 ± 1.22	18.22 ± 1	18.09 ± 0.94	17.6 ± 1.18
	SX-L	18.77 ± 0.42	18.44 ± 0.76	18.03 ± 0.97	17.79 ± 0.84	17.53 ± 0.9	17.22 ± 1.03	16.53 ± 1.24
	ISD	18.45 ± 0.91	18.22 ± 0.71	18.04 ± 0.56	18.17 ± 0.96	17.77 ± 0.95	17.74 ± 0.77	17.61 ± 1.41
O_2_ Extr (%)	Sham	0.36 ± 0.22	0.37 ± 0.21	0.37 ± 0.21	0.5 ± 0.25	0.5 ± 0.2	0.58 ± 0.21	0.58 ± 0.21
	Model	0.42 ± 0.14	0.47 ± 0.19	0.53 ± 0.21	0.59 ± 0.17	0.67 ± 0.13	0.66 ± 0.16	0.71 ± 0.15
	SX-H	0.36 ± 0.17	0.39 ± 0.24	0.43 ± 0.13	0.47 ± 0.12	0.54 ± 0.09*	0.55 ± 0.13*	0.59 ± 0.11*
	SX-M	0.33 ± 0.2	0.35 ± 0.22	0.42 ± 0.23	0.46 ± 0.23	0.51 ± 0.23	0.51 ± 0.25	0.53 ± 0.28
	SX-L	0.36 ± 0.16	0.48 ± 0.19	0.53 ± 0.16	0.51 ± 0.22	0.51 ± 0.16	0.57 ± 0.11	0.64 ± 0.15
	ISD	0.35 ± 0.07	0.37 ± 0.09	0.36 ± 0.13	0.43 ± 0.1	0.44 ± 0.14*	0.45 ± 0.17*	0.51 ± 0.2*
MVO_2_ (ml/min · 100 g)	Sham	9.72 ± 5.64	10.15 ± 4.05	9.95 ± 4.4	13.95 ± 7.14	15.5 ± 8.73	17.94 ± 9.83	18.52 ± 7.22
	Model	10.79 ± 5.28	15.53 ± 10.01	16.58 ± 8.45	19.24 ± 7.82	20.64 ± 7.62	23.54 ± 8.18	26.36 ± 6.74
	SX-H	9.92 ± 4.5	11.05 ± 7.99	10.81 ± 2.06	13.36 ± 3.85	15.75 ± 5.73	16.16 ± 7.54	19.8 ± 5.45
	SX-M	7.79 ± 2.51	9.13 ± 4.83	9.36 ± 2.39	9.93 ± 3.8	13.05 ± 7.04	16.47 ± 10.73	18.03 ± 8.71
	SX-L	8.75 ± 5.14	11.31 ± 3.73	12.44 ± 2.06	13.67 ± 5.81	14.82 ± 4.77	15.62 ± 3.61	17.05 ± 4.04
	ISD	11.7 ± 1.36	13.59 ± 4.41	11.18 ± 3.09	14.51 ± 2.54	14.31 ± 3.46	16.22 ± 5.67	21.92 ± 12.7

All data were expressed as mean ± S.D. *CvO*_*2*_ oxygen content of venous, *CaO*_*2*_ oxygen content of arterial, *O*_*2*_ *Extr* oxygen extraction ratio, *MVO*_*2*_ myocardial oxygen consumption. *P < 0.05 vs. model group

## Discussion

Previous clinical research indicates that, SX has been shown to cause rapid remission of angina pectoris and no serious side effects [30]. In our study, we demonstrated that SX can reduce myocardium injuries after occlusion of the LAD in anaesthetized dog. And the present data demonstrate that: 1) SX can obviously reduce the levels of cardiac enzyme CK-MB, LDH and MDA and increase the activity of SOD; 2) SX can markedly reduce the myocardial infarct size, the degree (∑-ST) and scope (N-ST) of myocardial ischemia; 3) SX can reduce MBP, LVSP, CVR and increased CBF, MBF; 4) SX can significantly reduce myocardial oxygen extraction ratio. The research methods and implications of these findings is discussed below.

In human disease, myocardial ischemia usually occurs in different patterns, this various patterns of ischemic myocardial necrosis occur for the most part against the background of a coronary circulation widely compromised by stenosing atherosclerosis [31, 32]. Conventional myocardial ischemia model is induced by ligating the left anterior descending artery. In fact this method actually reflects myocardial infarction and it did not fully represent disease characteristics of coronary heart disease, angina, and ultra-early clinical myocardial infarction. Because the early stage of these disease in clinical state were incomplete occlusion whereas the model of myocardial infarction was completely occulted. Moreover the drug hardly passes and plays its role in the complete occlusion model. Therefore, this study attempts to establish acute myocardial ischemia model by incomplete ligating the left anterior descending artery to reduce flow by 90 %, and evaluate the protective effect of drugs on early myocardial ischemia. The animal species of this research was dog because of that many factors such as heart size, beating rate, and molecular composition were more closer to human than with small animals [33].

The technique of epicardial ECG was used in our studies to delineate the area of ischemia. Epicardial ECG ST-segment analysis in the characterization of myocardial ischemia and infarction has received wide application [34]. This technique can accurately evaluate the extent and magnitude of ischemic injury following coronary occlusion and appears to be an indicator of myocardial ischemia [35, 36]. There is considerable evidence suggesting that the epicardial S-T segment elevation directly reflects myocardial cellular injury. Ross [37] reported that epicardial ECG analysis was useful for detecting acute changes in the severity of ischemic injury, and correlated with biochemical changes, regional myocardial blood flow, histologic changes, and creating phosphokinase (CPK), myocardial electrolyte alterations. Our data indicated that SX could markedly lower the degree of myocardial ischemia (Σ-ST) and reduce the scope of ischemia (N-ST) in dogs, which was in accordance with the result of reducing infarction and other specific parameters of ischemic damage.

Previous studies showed that acute myocardial ischemia can create numerous free radicals that cause damage to the cellular membranes as a result of producing lipid peroxidation [38]. When cell membrane becomes permeable or rupture, LDH, and CK spread out from the damaged tissues to the blood stream and served the diagnostic markers of myocardial tissue injury [39]. MDA was diagnostic indices of lipid peroxidation and peroxidative tissue injury [40, 41]. However, SOD enzyme can protect the cell from superoxide toxicity through catalyzing the conversion of superoxide free radicals to oxygen and hydrogen peroxide in all cells [42]. Our data showed that the levels of MDA, LDH, and CK-MB were decreased and SOD was increased significantly in SX administered group, suggesting that SX may reduce lipid peroxidation resulting in the protection against the myocardial ischemic damage.

In this study, SX increased coronary blood flow substantially and decreased arterial pressure slightly as coronary vascular resistance was reduced. Since heart rate and myocardial contractility (dP/dtmax) was held constant, the increase in coronary blood flow was due to an augmentation in stroke volume and expansion of the coronary arteries. After myocardial, cardiac preload, and afterload gradually increased eventually leading to more debilitating heart function [43, 44]. LVEDP and CVR were used as indicators of cardiac preload and afterload [45]. The decrease in preload (LVEDP, by −31.4 %) and afterload (CVR, by −56.1 %) of SX high dose group suggested the beneficial effect of SX in reducing cardiac load.

Other previous researches suggested that, a rapid increase of myocardial oxygen demand occurred in ischemia, and influence myocardial oxygen demand may aggravate or alleviate symptoms of myocardial ischemia [46]. The balance between oxygen supply and demand is of paramount importance for the heart since it determines where oxygen need exceeds oxygen supply [47]. Clinical observations suggested that the associated reduction of myocardial oxygen needs is often associated with relief of angina in patients with angina treatment of the hypermetabolic state [46]. Reducing myocardial oxygen consumption produced potent anti-ischemic effects [48]. In the current study, a significant decreasing of myocardial oxygen extraction was found in groups treated with SX, and the same trend was observed in myocardial oxygen consumption.

## Conclusions

The present research suggests that SX exert notable anti-anginal ischaemia effect, and indicates SX as an effective traditional Chinese medicine for the treatment of ischemic heart disease. The mechanism of actions related to SX can improve hemodynamics, expand coronary artery, and reduce myocardial workload and oxygen demand.

## Abbreviations

SX: Suxiaojiuxin Pill
ISD: Isosorbide dinitrate
CHD: Coronary heart disease
AMI: Acute myocardial ischemia
TCM: Traditional Chinese medicine
LAD: Left anterior descending
LCX: Left circumflex coronary artery
LDH: Lactate dehydrogenase
CK: Creatine kinase
MDA: Malondialdehyde
SOD: Superoxide dismutase
HR: Heart rate
Σ-ST: Total mV of ST segment elevation
N-ST: Total number of sites with this degree of ST segment elevation
MAP: Mean arterial pressure
LVSP: Left ventricular systolic pressure
LVEDP: Left ventricular end-diastolic pressure
+dP/dtmax: Maximal positive left ventricular dP/dt
-dP/dtmix: Minimal positive left ventricular dP/dt
CBF: Coronary blood flow
MBF: Myocardial blood flow
CVR: Coronary vascular resistance
CvO_2_: Oxygen content of venous
CaO_2_: Oxygen content of arterial
O_2_ Extr: Oxygen extraction ratio
MVO_2_: Myocardial oxygen consumption

## References

[1] Finegold JA, Asaria P, Francis DP (2013). Mortality from ischaemic heart disease by country, region, and age: statistics from World Health Organisation and United Nations. Int J Cardiol.

[2] Fuster V, Kelly BB (2010). Promoting cardiovascular health in the developing world: a critical challenge to achieve global health. Washington (DC).

[3] Yellon DM, Hausenloy DJ (2007). Myocardial reperfusion injury. N Engl J Med.

[4] Qiu Y, Xu H, Shi D (2012). Traditional Chinese herbal products for coronary heart disease: an overview of Cochrane reviews. Evid Based Complement Alternat Med.

[5] Fan FF, Xu Q, Sun Q, Zhao SJ, Wang P, Guo XR (2014). Assessment of the reporting quality of randomized controlled trials on treatment of coronary heart disease with traditional Chinese medicine from the Chinese Journal of Integrated Traditional and Western Medicine: a systematic review. PLoS One.

[6] O’Brien K, Luis Vitetta L. The potential role of herbal medicines in the treatment of chronic stable angina pectoris: a review of key herbs, and as illustration, exploration of the Chinese herbal medicine approach. Botanics: Targets and Therapy. 2012;1.

[7] Pu L (2006). Suxiao Jiuxin Pills and new drug–chuanxiong, Borneolum. Beijing Journal of Traditional Chinese Medicine.

[8] Duan KJ, Zhang CY, Yang XY (2002). Clinical observation on 40 cases of coronary heart disease and angina pectoris treated by Suxiao Jiuxin Wan. Tianjin Journal of Traditional Chinese Medicine.

[9] Li CS, Qu ZQ, Wang SS, Hao XW, Zhang XQ, Guan J, Han F (2011). Effects of suxiao jiuxin pill on oxidative stress and inflammatory response in rats with experimental atherosclerosis. J Tradit Chin Med.

[10] Cao SH, Yan XX, Zhang JY, Zhang GM, Zhang YY (2007). Clinical observation on 105 short-term treatment of coronary heart disease and angina pectoris treated by suxiao jiuxin wan. Chinese Traditional Patent Medicine.

[11] Zhao W, Bi HG, Zhang KN, Wang M, Zhang LH (2005). The clinical research on activating blood to resolve stasis of Su Xiao Jiu Xin Wan. Chinese Journal of Medical Physics.

[12] Zhang J, Zhuang P, Lu Z, Zhang M, Zhang T, Zhang Y, et al. Suxiaojiuxin pill enhances atherosclerotic plaque stability by modulating the MMPs/TIMPs balance in apoE-deficient mice. J Cardiovasc Pharmacol. 2014. 10.1097/FJC.0000000000000095 24621651

[13] Xie D, Sheng J (2013). Clinical research progress of Suxiao Jiuxin pills treatment of angina pectoris of coronary heart disease. China Journal of Chinese Medicine.

[14] Bai X, Zhang P, Yang Q, Liu X, Wang J, Tong Y, Xiong S, Liu L, Wang L, He G (2014). Suxiao Jiuxin pill induces potent relaxation and inhibition on contraction in human artery and the mechanism. Evid Based Complement Alternat Med.

[15] Wang B, Ni Q, Wang X, Lin L (2012). Meta-analysis of the clinical effect of ligustrazine on diabetic nephropathy. Am J Chin Med.

[16] Zhao Z, Yu H, Lu K, Yang S (1991). Determination of tetramethyl pyrazine in traditional Chinese medicines by high performance liquid chromatography. Zhongguo Zhong Yao Za Zhi.

[17] Xiong ZY, Xiao FM, Xu X, Wu YF, Jiang XM (2013). [Studies on pharmacological activity of borneol]. Zhongguo Zhong Yao Za Zhi.

[18] Xu P, Li Y, Du SY, Lu Y, Bai J, Guo QL (2014). Comparative pharmacokinetics of borneol in cerebral ischemia-reperfusion and sham-operated rats. J Zhejiang Univ Sci B.

[19] Regulations on the management of laboratory animal. Gazette of the State Council of the People’s Republic of China 1988(25):823–827.

[20] Du LL, Gao ZG, Nithipatikom K, IJzerman AP, van Veldhoven JPD, Jacobson KA, Gross GJ, Auchampach JA (2012). Protection from myocardial ischemia/reperfusion injury by a positive allosteric modulator of the A(3) adenosine receptor. J Pharmacol Exp Therapeut.

[21] Auchampach JA, Ge ZD, Wan TC, Moore J, Gross GJ (2003). A3 adenosine receptor agonist IB-MECA reduces myocardial ischemia-reperfusion injury in dogs. Am J Physiol Heart Circ Physiol.

[22] Ansorge EJ, Augustyniak RA, Perinot ML, Hammond RL, Kim JK, Sala-Mercado JA, Rodriguez J, Rossi NF, O’Leary DS (2005). Altered muscle metaboreflex control of coronary blood flow and ventricular function in heart failure. Am J Physiol Heart Circ Physiol.

[23] Verrier RL, Pagotto VP, Kanas AF, Sobrado MF, Nearing BD, Zeng D, Belardinelli L (2013). Low doses of ranolazine and dronedarone in combination exert potent protection against atrial fibrillation and vulnerability to ventricular arrhythmias during acute myocardial ischemia. Heart Rhythm.

[24] Guangxian C, Yuhong W (2010). Protective effect of Xinning tablet on acute myocardial ischemia in anesthesia dogs. World J Sci Tech.

[25] Range FT, Schafers M, Acil T, Schafers KP, Kies P, Paul M, Hermann S, Brisse B, Breithardt G, Schober O (2007). Impaired myocardial perfusion and perfusion reserve associated with increased coronary resistance in persistent idiopathic atrial fibrillation. Eur Heart J.

[26] Fu JH, Zheng YQ, Li P, Li XZ, Shang XH, Liu JX (2013). Hawthorn leaves flavonoids decreases inflammation related to acute myocardial ischemia/reperfusion in anesthetized dogs. Chin J Integr Med.

[27] Liu JX, Li XZ, Cong WH, Fu JH, Shang XH (2007). Effects of Corocalm (Shuguan Capsule) on acute myocardial ischemia in anesthetized dogs. Chin J Integr Med.

[28] Taverne YJ, de Beer VJ, Hoogteijling BA, Juni RP, Moens AL, Duncker DJ, Merkus D (2012). Nitroso-redox balance in control of coronary vasomotor tone. J Appl Physiol.

[29] Zhao ZQ, Corvera JS, Halkos ME, Kerendi F, Wang NP, Guyton RA, Vinten-Johansen J (2003). Inhibition of myocardial injury by ischemic postconditioning during reperfusion: comparison with ischemic preconditioning. Am J Physiol Heart Circ Physiol.

[30] Duan X, Zhou L, Wu T, Liu G, Qiao J, Wei J, Ni J, Zheng J, Chen X, Wang Q (2008). Chinese herbal medicine suxiao jiuxin wan for angina pectoris. Cochrane Database Syst Rev.

[31] Woolf N (1977). Animal models of myocardial ischaemia. J Clin Pathol.

[32] Libby P, Theroux P (2005). Pathophysiology of coronary artery disease. Circulation.

[33] Coppola B, Omens J (2008). Use of larger species such as dog and pig as model systems to study cardiac disease. Drug Discov Today Dis Model.

[34] Cohn LH, Lamberti JJ, Florian A, Moses R, Vandevanter S, Kirk E, Collins JJ (1975). Effects of hemodilution on acute myocardial ischemia. J Surg Res.

[35] KJEKSHUS JK, MAROKO PR, SOBEL BE (1972). Distribution of myocardial injury and its relation to epicardial ST-segment changes after coronary artery occlusion in the dog. Cardiovasc Res.

[36] Shouzhu H: Methods of treatment of cardiovascular and cerebrovascular diseases with fucoidan. Google Patents; 2009

[37] Ross J (1976). Electorcardiographic ST-segment analysis in the characterization of myocardial ischemia and infarction. Circulation.

[38] Rao PS, Cohen MV, Mueller HS (1983). Production of free radicals and lipid peroxides in early experimental myocardial ischemia. J Mol Cell Cardiol.

[39] Mehdizadeh R, Parizadeh MR, Khooei AR, Mehri S, Hosseinzadeh H (2013). Cardioprotective effect of saffron extract and safranal in isoproterenol-induced myocardial infarction in Wistar rats. Iranian Journal Of Basic Medical Sciences.

[40] Marnett LJ (2002). Oxy radicals, lipid peroxidation and DNA damage. Toxicology.

[41] Gaweł S, Wardas M, Niedworok E, Wardas P (2003). [Malondialdehyde (MDA) as a lipid peroxidation marker]. Wiadomosci lekarskie (Warsaw, Poland: 1960).

[42] Bagis S, Tamer L, Sahin G, Bilgin R, Guler H, Ercan B, Erdogan C (2005). Free radicals and antioxidants in primary fibromyalgia: an oxidative stress disorder?. Rheumatol Int.

[43] Jamshidi P, Kobza R, Toggweiler S, Arand P, Zuber M, Erne P (2012). Impact of preload changes on positive and negative left ventricular dP/dt and systolic time intervals: preload changes on left ventricular function. Indian Heart J.

[44] Feng J, Schaus BJ, Fallavollita JA, Lee TC, Canty JM (2001). Preload induces troponin I degradation independently of myocardial ischemia. Circulation.

[45] Thormann J, Schlepper M, Kramer W, Gottwik M, Kindler M (1983). Effectiveness of nicorandil (SG-75), a new long-acting drug with nitroglycerin effects, in patients with coronary artery disease: improved left ventricular function and regional wall motion and abolition of pacing-induced angina. J Cardiovasc Pharmacol.

[46] Maroko PR, Kjekshus JK, Sobel BE, Watanabe T, Covell JW, Ross J, Braunwald E (1971). Factors influencing infarct size following experimental coronary artery occlusions. Circulation.

[47] Zuurbier CJ, van Iterson M, Ince C (1999). Functional heterogeneity of oxygen supply-consumption ratio in the heart. Cardiovasc Res.

[48] Colin P, Ghaleh B, Monnet X, Hittinger L, Berdeaux A (2004). Effect of graded heart rate reduction with ivabradine on myocardial oxygen consumption and diastolic time in exercising dogs. J Pharmacol Exp Ther.

